# Notes and News

**Published:** 1901-01-12

**Authors:** 


### NOTES AND NEWS.

Smyrna is still under suspicion on account of rumours of fresh cases of the plague, and Turkish and Greek ports have established a ten days' quarantine for vessels arriving thence.

Statistics show that the modes of life adopted by Quakers is favourable to longevity. The rate of death amongst the very old and the very young is more especially below the average of the general population.

Amongst the beneficiaries by Lord Armstrong's will are numbered the Newcastle Hospital for Sick Children, the Blind Asylum, the Deaf and Dumb Institution, the Eye Infirmary, and the Convalescent Home at Whitley.

The great risk which the public runs in using tinned foods was illustrated by the revelations at Brighton last week, when an Italian firm of provision importers were shown to have 247 tins of utterly bad vegetables, all of which had been opened to let the bad gases out and again resoldered.

Although it is a long time since the epidemic of typhoid broke out in Belfast, the fever still continues prevalent, and has appeared where dwellings and soil were regarded as satisfactory. Attention is now being focussed upon the milk supply, and extensive arrangements for sterilising are contemplated.

Discoverers of the true elixir of life, otherwise a solution of common salt, are struggling for the honour of being regarded as the first in the field of discovery. One of the claimants is an English nurse, on whose behalf it is stated that American patients may have carried the report of the discovery to America, whence the boom is now spreading.

The New York Hospital has been considerably enlarged, and the new buildings have just been opened. The largest among them is the building for the private patients, which is ten stories high. As it is equipped with modern elevators the upper rooms are said to be even more desirable than the lower ones. Many of the rooms are arranged in suites for those who desire complete accommodation, but there are a large number of single rooms for those who need privacy at a moderate outlay.

A Cottage Hospital has been presented to Moreton Hampstead, in Devonshire, by the Hon. W. Smith, who has a residence near.

It is strange that with the adoption of the open-air cure for consumption that from therapeutic remedies are being brought forward also.

The Men's Convalescent Institution at Rhyl now possesses 122 beds, and has done good work during the past year. For several months every bed was occupied.

It is stated that two old men, inmates of almhouses in New York, are undergoing a course of glycero-phosphate of sodium treatment to test the efficacy of the new elixir.

Alderman Davies of Lancaster, who was Mayor of Preston during the Jubilee year, has given £2,000 to the Preston Infirmary, to establish a memorial to his son, who died in South Africa whilst acting as civil surgeon with the Army.

Mr. Edward Trimmer has retired from the secretary ship of the Royal College of Surgeons, after forty-two years' service. Mr. Trimmer was both active and influential in the affairs of the College, and he will be much missed both personally and officially.

Aberdeen has been the recipient of immense generosity recently. Lord Mountstephen's gift to the Royal Infirmary to clear it from debt amounted to £25,000. Lord Strathcona has made a promise of £25,000 to complete the plans of the University if £50,000 is raised during the present year, Mr. Charles Mitchell, of Newcastle, having undertaken to defray the present debt on the University, amounting to £20,000.

The sacred necessity of comfort is one of the key-notes of the present century. It will afford fresh opportunities for expenditure in every direction as the standard rises. The Aylesbury Workhouse has begun the new century in truly modern spirit, the inmates are to be made more comfortable by the introduction of wider beds. Probably other workhouses could be improved in this respect, and will have to follow suit.

The subject of visitors to hospitals is being discussed at present in the lay press. It appears that an article on the subject which was published in one paper inspired several well-meaning ladies to immediately rush to the rescue of the hospitals. Since then their experiences have been appearing in print. They have evidently not received the warm welcome at the hands of the authorities which they expected. Sometimes their reception was apparently not very courteous. This of course we do not defend, but we feel sure that at all really well conducted hospitals any offers of useful co-operation will not be rejected. The difficulty is that many ladies think their kind hearts will inspire them with all the necessary qualities for visiting the sick in hospital wards. Those within know how undesirable the injudicious visitor is. Also, if the patients could speak quite frankly, many a kind visitor would hear how little interest the company of such a stranger affords. There is quite enough life within a hospital ward to occupy the attention of the majority of the sick, and the visiting day brings to most the real interest with the friends from without.

